# A Rare Presentation of Cystic Metastatic Melanoma in the Upper Chest

**DOI:** 10.1155/crdm/8706831

**Published:** 2025-10-28

**Authors:** Madeline Tchack, Noah Musolff, Sydney Wolfe, Ria Sandeep, Babar Rao

**Affiliations:** ^1^Rutgers Robert Wood Johnson Medical School, Piscataway, New Jersey, USA; ^2^Center for Dermatology, Rutgers Robert Wood Johnson Medical School, Somerset, New Jersey, USA; ^3^Rao Dermatology, New York, New York, USA; ^4^Georgetown School of Medicine, Washington, DC, USA; ^5^Department of Dermatology, Weill Cornell Medicine, New York, New York, USA

**Keywords:** cystic metastatic melanoma, MART-1, melanoma of unknown primary, noncutaneous primary melanoma, SOX-10

## Abstract

A 57-year-old male was evaluated for a smooth cystic nodule on his left upper chest that appeared clinically to be a cyst, yet histopathology and confirmatory immunohistochemistry revealed invasive malignant melanoma. Histopathologic evaluation and immunohistochemical staining (i.e., SOX-10 and MART-1) are useful diagnostic tools for invasive malignant melanoma that, albeit rare, can present without known or cutaneous primary. This case illustrates the variability with which metastatic melanoma can present, calling attention to the importance of careful evaluation of benign-appearing and asymptomatic lesions.

## 1. Introduction

A diagnostic challenge to dermatologists, malignant melanoma can present variably, ambiguously, benignly, or even metastatically with or without evidence of a known primary. Thus, it is important to consider the possibility of asymptomatic and/or benign-appearing lesions to be malignant and to correlate clinical findings with histopathology and confirmatory immunohistochemical staining.

## 2. Case Report

**Figure 1:**
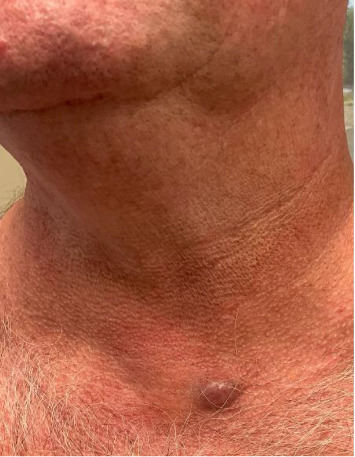
Clinical examination showed a smooth cystic nodule on the left upper chest (1.8 × 1.8 cm).

A 57-year-old male presented to the clinic for a full skin examination and was found to have a 1.8 × 1.8 cm smooth cystic nodule on his left upper thorax just inferior to the neck and lateral to the sternum (Figure 1) that had been present for the past four months and causing discomfort. The patient had a remarkable dermatologic history of nonmelanoma skin cancer and active rosacea on the face. His medical history was remarkable for diabetes, hypertension, hypercholesterolemia, and active alcohol abuse. Multiple common nevi, solar lentigines, seborrheic keratoses, and hemangiomas were also noted at the time of his full skin examination, but no other suspicious lesions were identified. The clinical diagnosis of epidermoid cyst was made and an excision measuring 1.5 × 1.5 cm was performed.

**Figure 2:**
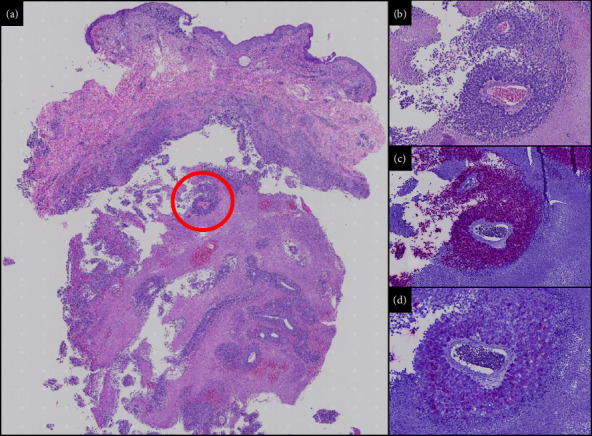
Histopathology at 400x magnification. (a) H&E: the image shows an intact epidermis with a focal underlying inflammatory infiltrate. In the deep dermis and subcutaneous tissue, a nodular lesion is present with diffuse nests of atypical cells. (b) Close-up of red circle in (a): diffuse atypical cells surrounding a dermal vessel. (c) SOX-10 staining of (b): positive staining of the atypical cells. (d) MART-1 staining of (b): positive staining of the atypical cells.

Histopathological examination featured a deep dermal nodule composed of a cystic space adjacent to a proliferation of atypical cells and marked necrosis extending into subcutaneous tissue without epidermal involvement (Figure 2(a) and 2(b)). Additional staining was performed demonstrating positive SOX-10 (Figure 2(c)) and focally positive MART-1 (Figure 2(d)) in atypical cells in deep dermal and subcutaneous tissues, confirming the diagnosis of invasive malignant melanoma. The patient was referred to oncology for further management.

## 3. Discussion

Clinically, malignant melanoma can be challenging to diagnose given its variable presentation. Recognizing and diagnosing some melanoma variants may be especially difficult given their rarity or resemblance to benign lesions [1] or metastatic melanoma, which may not always present with an obvious site of origin, cutaneous or otherwise [2–4]. Most cases of melanoma are cutaneous with a known primary site; however, ∼5% of melanomas are noncutaneous primary melanomas (NPM) [5] and 1%–8% of cases are melanomas with unknown primary (MUP) [3, 4]. NPM typically present as ocular melanoma (73%); mucosal melanoma (27%) [6] in the sinonasal, gastrointestinal, or genitourinary tracts [5]; or, very rarely, in the adrenal glands [6]. MUP are metastatic malignant melanomas that present without evidence of a clear site of origin and are often discovered as secondary deposits in lymph nodes, subcutaneous tissues, or other organs [3, 4]. Though several hypotheses exist, most believe that MUP result from immune-mediated primary tumor regression after metastasis has occurred [3, 7].

The skin itself is the most common site of melanoma metastasis (56%) from any melanoma subtype, and in 5% of the patients, metastatic melanoma is the initial presentation of disease [2, 8]. Over 90% of melanoma metastasis presents as a single dermal nodule but occasionally resembles benign, cystic, infectious, inflammatory, or autoimmune skin lesions [8–10], while rare, cystic metastasis of malignant melanoma most frequently occurs in the brain in the context of widespread metastatic disease [11, 12]. One reported case of cystic metastatic malignant MUP presented as a cystic mass in the anteromedial compartment of the thigh; however, no cases of cystic metastasis secondary to NPM have been reported [13]. To our knowledge, the patient described herein is the first reported case of cystic metastatic melanoma in the upper chest from either a NPM or MUP. In cases of rare, atypical, ambiguous, or advanced disease, clinical correlations to histopathology and immunohistochemistry are often critical in accurately distinguishing benign lesions from nonmelanoma skin cancers, primary cutaneous melanoma, melanoma metastases, or other rare melanoma variants to appropriately guide further management.

Cystic metastasis of malignant melanoma reportedly appears as uni- or multilocular cyst-like cavities filled with necrotic material [13]. In this case, cystic spaces and atypical cells with marked necrosis on initial histopathological evaluation raised suspicion of melanoma, prompting additional examination with immunohistochemistry. Traditionally, S100 has been the immunohistochemical marker of choice in identifying nodal melanoma metastasis due to high sensitivity for melanocytes; however, other cell types (e.g., dendritic cells and Langerhan's cells) may also test positive for S100, potentially hindering accurate identification of micrometastasis and melanoma tumor cells [14, 15]. SOX-10, a transcription factor that plays an important role in melanocytic development but is not expressed in dendritic cells, is notably a sensitive diagnostic indicator of nodal melanoma metastasis and has especially improved the identification of micrometastasis compared with other widely used immunostains, such as S100, Melan-A/MART-1, and HMB-45 [14, 16, 17]. Other studies investigating the efficacy of staining using Melan-A/MART-1 antibodies compared with S100 found that they demonstrated specific staining of melanocytes with minimal cross-reactivity for other cells while unequivocally recognizing micrometastasis in sentinel lymph nodes.^15^ While our immunohistochemical staining was performed on the cutaneous sample itself and was not derived from lymph nodes, one study suggests that consecutive staining of MART-1 followed by SOX-10 can be applied to detect melanocytes in both sentinel lymph nodes and skin tissue [18]. Moreover, their evidence supports that positive staining of both SOX-10 and MART-1 suggests the diagnosis of melanoma.

Histologically, cutaneous melanoma metastasis (CMM) commonly appears as well-circumscribed nodular aggregates of atypical melanocytes confined to the dermis without epidermal involvement, while primary cutaneous melanoma usually demonstrates atypical melanocytes involving the epidermis [2]. CMM is often indistinguishable histologically from primary dermal melanoma (PDM), a primary melanoma tumor confined to the dermis and/or subcutis without epidermal involvement [19], which presents as a firm subcutaneous nodule that may be skin-colored [20] or have features of subcutaneous cystic or vascular processes or nonmelanoma skin cancers [21]. A case of PDM reported by Weiss et al. noted positive staining for SOX-10 but negative staining for Melan-A/MART-1, suggesting a potential distinction between, otherwise, histologically similar PDM and CMM [19]. In our case, H&E revealing atypical cells in a deep dermal nodule confirmed to be melanocytes with positive staining of SOX-10 and MART-1 likely favors a diagnosis of melanoma metastasis over a primary cutaneous melanoma given the absence of positive staining in the epidermis.

Though cystic lesions infrequently resemble cases of melanoma metastasis clinically, benign-appearing cysts have, on rare occasion, turned out to be malignant as in the patient reported herein. Epidermoid cysts usually arise from the infundibular epithelium of the hair follicle [22] on the face, back, or chest [23] and, while benign, can occasionally undergo malignant transformation into squamous cell carcinoma [24] or involve inflammatory dermatoses, infectious entities, or malignant tumors in the epithelial linings [25]. Swygert et al. described a case of melanoma in situ involving an epidermoid cyst [25] and emphasized the importance of presumed cyst removal and subsequent histopathologic examination of the surrounding epithelium for neoplastic processes. Our case together with prior literature suggest that malignant neoplasms should be considered as part of the differential diagnoses of benign-appearing, cystic lesions instead of being overlooked or being removed electively only for cosmetic purposes.

Though our patient was referred to oncology for further management and is no longer being followed, incomplete excision of the original lesion in addition to its atypical presentation, unknown site of origin, and unknown extent of metastasis are all reasons to consider re-excision as part of the patient's management to minimize the possibility of residual disease at the site of the cyst. Furthermore, because neither MUP nor NPM have been ruled out and the extent of disease is unknown, a more extensive skin evaluation, an ophthalmic evaluation, imaging (CT chest/abdomen/pelvis and MRI brain) to complete staging, and molecular testing (e.g., BRAF and NRAS) should be pursued in anticipation of systemic therapy [4]. Without this additional workup, it is not possible to clearly distinguish whether this case represents MUP, which requires exhaustive negative evaluation, or NPM, which is typically confirmed by identifying an ocular or mucosal origin. The unknown outcome of such testing (if any was performed) remains a key limitation of this report. If the patient was lost to oncologic follow-up, this could significantly hinder accurate staging and continuity of care, ultimately limiting therapeutic options that are essential for prognosis and management.

In conclusion, this case report highlights the difficulties in diagnosing a rare case of cystic metastatic melanoma and distinguishing it from primary cutaneous melanoma, other rare melanoma variants, or benign cystic lesions. This challenge emphasizes the importance of correlating clinical findings to histopathology and confirmatory immunohistochemical staining (i.e., SOX-10 and MART-1) and underscores the need to consider the potential for malignancy in asymptomatic and/or benign-appearing cases. In circumstances where a primary cutaneous lesion cannot be identified, both MUP and NPM should be considered, among other tumors, prompting further workup with a more extensive skin evaluation, an ophthalmic evaluation, general imaging, and molecular testing.

## Data Availability

Data sharing is not applicable to this article as no datasets were generated or analyzed during the current study.
